# To the Editor— Functional substrate mapping of ventricular arrhythmia

**DOI:** 10.1016/j.hroo.2024.04.015

**Published:** 2024-11-22

**Authors:** Henry H. Hsia, Won-Seok Choe

**Affiliations:** Arrhythmia Service, University of California, San Francisco, San Francisco, California

We read with great interest the article by Vlachos et al.^1^ Because of the high incidence of nonhemodynamically tolerated ventricular arrhythmias, the recent paradigm shift has led to the development of mapping strategies during sinus rhythm. This article provided an excellent review of various high-density signal processing algorithms and mapping techniques for substrate characterization.

There is increasing evidence that critical parts of the arrhythmia circuits are formed by functional rather than fixed lines of block.^2^ Multiple reentry morphologies with shared isthmus often arise with reentrant circuits overlapping the same general area. However, electrophysiological parameters that are critical for the initiation and maintenance of reentry may be present during baseline rhythm, dictated by the local myocardial topographies and/or nonuniform anisotropic conduction properties.^3^ Such features can be detected by functional mapping strategies that include isochronal late activation mapping,^4^ decremental evoked potential,^5^ and hidden slow conduction.^6^

We wish to comment on Figures 1 and 3, which were adapted from the original article by Srinivasan et al^7^ using a sensed protocol for dynamic functional mapping. We believed the figures were reversed. The value of electrogram morphology to identify the active circuit (pages 137–138) should be referenced in Figure 3, while functionally guided substrate modification approaches (sensed protocol) (pages 139–140) were described in Figure 1. In addition, the exit vs entrance sites were reversed in Figure 3. This was supported by the corresponding electrocardiographic QRS morphology and the online supplemental video from the original publication, which suggested that the exit was located at the anterosuperior base of the left ventricle.

Figures 3A–C described pace mapping at the isthmus site (see text), but the arrows showed pacing during reentry, with an antidromic collision near the entrance and orthodromic propagation out of the exit. Figures 3D and 3E depicted the electrogram morphologies at various locations of the putative circuit in sinus rhythm. The wavefront propagation during sinus rhythm should proceed along the lateral isthmus boundaries with conduction delay/block, either anatomical or functional, resulting in late potentials recorded at the isthmus and fractionated local abnormal ventricular activities near the entrance and exit sites. However, the arrow were confusing and should be revised.

Functional substrate mapping unmasks the critical components of the reentrant circuit(s), allows more targeted ablation procedures, and potentially shortens the procedural time, particularly in unstable patients with limited cardiac reserve and large substrates. However, a number of issues need to be addressed: (1) What is the appropriate wavefront activation relative to the location of the arrhythmia circuit?^8^ (2) How to achieve a streamlined, efficient workflow during mapping? (3) How to optimize signal processing and develop automated annotation algorithms. A standardized and objective approach to delineate fixed and functional components of substrates without arrhythmia induction is essential to further improve ablation outcomes across centers.
